# Evaluation of practice nurses’ management of paediatric psychosocial problems in general practice

**DOI:** 10.1093/pubmed/fdae008

**Published:** 2024-01-31

**Authors:** Lukas B M Koet, Heike Gerger, Wilma Jansen, Patrick J E Bindels, Evelien I T de Schepper

**Affiliations:** Department of General Practice, Erasmus MC, University Medical Centre Rotterdam, 3000 CA Rotterdam, the Netherlands; Department of General Practice, Erasmus MC, University Medical Centre Rotterdam, 3000 CA Rotterdam, the Netherlands; Department of Clinical Psychology, Open University, 6419 AT Heerlen, the Netherlands; Department of Public Health, Erasmus MC, University Medical Centre Rotterdam, 3000 CA Rotterdam, the Netherlands; Department of Youth, City of Rotterdam, Rotterdam, The Netherlands; Department of General Practice, Erasmus MC, University Medical Centre Rotterdam, 3000 CA Rotterdam, the Netherlands; Department of General Practice, Erasmus MC, University Medical Centre Rotterdam, 3000 CA Rotterdam, the Netherlands

**Keywords:** adolescent, child, general practice, nurse specialists, mental disorders

## Abstract

**Background:**

Child mental health services are under major pressure worldwide. In the Netherlands, Youth Mental Health Practice Nurses (YMHPNs) have been introduced in general practice to improve access to care. In this study, we evaluated care delivered by YMHPNs.

**Methods:**

We used medical records of a population-based cohort (21 717 children, 0–17 years). Characteristics of children consulting a YMHPN, type of problem, care delivered by YMHPNs and referrals were assessed using quantitative content analysis.

**Results:**

Records of 375 children (mean age 12.9 years, 59.2% girl) were analysed. These children were often in their adolescence (57.3% was between 13 and 17 years), and more often female than male (59.2% vs 40.8%). YMHPNs had a median of four consultations (IQR 2–7) with the child. YMHPNs managed a variety of psychosocial problems. YMHPNs managed 22.4% of children without need of referral, 52.0% were eventually referred for additional care. 13.3% of children dropped out during the treatment trajectory. In the remaining 12.3% of children, the treatment trajectory was stopped because the child was already attending specialized services, the treatment trajectory was still ongoing or the medical record was inconclusive.

**Conclusions:**

YMHPNs successfully managed one in four children with psychosocial problems without need for referral. Nevertheless, most children were eventually referred for additional care.

## Introduction

Worldwide, child mental health services have been under much strain for years.^1^^,^^2^ A lack of resources and trained professionals result in long waiting-lists and rejection of referrals.^3^ These form major barriers to appropriate care for children and adolescents with mental health problems.^4^ In fact, even in high-income countries only a minority of children and adolescents with mental health problems attend mental health services.^5–8^ The recent COVID-19 pandemic has negatively affected the mental health of children,^9^^,^^10^ and it seems to have led to an increase in the demand for child mental health services.^11^^,^^12^

In the Netherlands, the use of youth care, including child mental health services, has increased strongly over the past two decades.^13^ In response, the Dutch youth care system has been reformed drastically in 2015 (Dutch Youth Act).^14^ In this new legislation, the organization and financing of youth care was transferred from national and regional governmental levels to the municipalities. This legislation aimed to make child mental health care more accessible and to improve integrated care (e.g. by implementing community-based support teams).^15^

Parallel to the Youth Act, a new position was introduced to integrate child mental health care into general practice: the Youth Mental Health Practice Nurse (YMHPN). YMHPNs are care professionals with a background in youth care (e**.**g**.** psychiatric nurse, psychologist, or social worker) working within general practices. YMHPNs provide a variety of tasks, such as problem clarification, support for problems concerning raising children, psycho-education, short-term treatment based on cognitive behavioural therapy and specific family interventions.^16^ Additionally, they support the GP with information and advice, act as the contact for external parties such as schools and specialized mental health services, and refer children in name of the GP if more specialized care is needed.^17^ YMHPNs are not licensed to prescribe medication. Consultations with a YMHPN usually take 30–60 minutes.^18^ Typically, there is no waiting list for the YMHPN.^19^^,^^20^

The financing of the YMHPN differs per practice, and is determined in consensus by GPs, municipalities, and insurance companies. Although exact numbers are lacking, the number of YMHPNs working in general practice has been growing steadily since 2015.^21^^,^^22^ GPs and patients have reported positively about the presence of YMHPNs in Dutch general practice.^18^^,^^20^ However, only limited information is available to date on the type of psychosocial problems managed by YMHPNs and how they exactly manage these problems.^16–18^^,^^23^^,^^24^

Therefore, we evaluated the care delivered by YMHPNs in-depth by investigating the electronic records of children consulting a YMHPN. We aimed to describe (1) the characteristics and the type of problems of children consulting YMHPNs and (2) the YMHPNs’ management of these problems.

## Methods

### Design

We used electronic records of children (0–17 years) in the Rijnmond Primary Care Database (RPCD), a region-specific derivative of the Integrated Primary Care Information database, focussed on the greater Rotterdam area. Rotterdam is the second largest city of the Netherlands (670,000 inhabitants), with a large community of ethnic minorities and the highest percentage of children living in a low-income household.^25^

The RPCD contains pseudonymised longitudinal medical data of general practice patients, such as symptoms, diagnoses, referrals, laboratory findings, drug prescriptions and specialists’ letters.^26^ Medical problems and diagnoses are coded with the International Classification for Primary Care 1 (ICPC-1).^27^^,^^28^ From the database, general practices exclusively located in the municipality of Rotterdam were selected that employ a YMHPN.

### Selection of relevant cases for medical record analysis

We developed a search-algorithm (see Supplementary Tables S1–S2) to detect medical records of children consulting a YMHPN for the first time between 1 July^t^ 2017 and 31 June 2021. Our algorithm selected the first date in the medical record of a child that contained either (1) free-text ‘practice nurse’ (including abbreviations) or (2) a specific insurance code used for care delivered by a practice nurse. The search algorithm automatically excluded records with less than 6 months of follow-up data after the first contact with the YMHPN, as we considered a minimum of 6 months of follow-up time as adequate for our research question to allow valid inferences on the treatment trajectories of the YMHPN. The search algorithm detected a total of 576 medical records. We extracted the date of contact, age at contact, sex, ICPC code (type of psychosocial problem) at contact.

### Analysis of the care delivered by YMHPNs

We studied the selected medical records using quantitative content analysis.^29^ We developed a checklist to describe and categorize the care delivered by the YMHPN, focussing on the first year after the YMHPN got involved with the patient (see Supplementary Table S3). To improve the reliability of the content analysis the first 50 cases were coded by two researchers and discussed case-by-case to improve subsequent coding. The following information was extracted manually by the first author from the medical record to describe care delivered during a treatment trajectory: number of consultations with a YMHPN; number of contacts of YMHPNs with external professionals; number of no shows; in case of referral, we recorded whether it was to (1) mental health services providing specialized mental health care or (2) to other care, defined as either a community-based support team or a centre for youth and family (CYF). A support team provides (social) help such as parenting support. A CYF is a regional institution with a focus on prevention. It monitors physical, psychosocial and cognitive development of children and also provides specific courses (e**.**g**.** social skills trainings). Both the support team and the CYF can refer children to specialized mental health services.

A treatment trajectory was defined as all care delivered by the YMHPN up to 1-year follow-up (ranging 6–12 months) from the moment the YMHPN got involved with the child. The following end-of-treatment trajectory were defined: (1) drop-out (ie, child/caregiver cancels treatment trajectory unilaterally or does not respond to request to make a new appointment); (2) child/caregiver and YMHPN decide together to end trajectory because no additional help is deemed necessary; (3) treatment trajectory ends in referral; (4) YMHPN stopped the treatment trajectory during intake because of no added value (i.e, it became clear that child was already receiving specialized care); (5) Treatment trajectory with YMHPN was not yet finished after 1-year follow-up; and (6) end-of-treatment trajectory is unclear from the record.

We aimed to extract the type of care delivered (e**.**g**.** psychoeducation, interventions based on cognitive-behavioural-therapy, etc.). However, in 128 of 375 records, YMHPNs used an additional electronic record for documentation of consultations which we had no access to. In these cases, the YMHPNs used the medical file of the GP only for recording summaries of the consultations but information on the used therapeutic approaches lacked. Therefore, we only extracted information on type of care from the 247 complete records. These 247 children did not differ substantially in characteristics from the overall group (see Supplementary Table S4).

### Statistics

We used descriptive statistics for the care delivered by YMHPNs by sex and age (young children: 0–6 years; school-age children: 7–12 years; adolescents: 13–17 years). Student’s *t*-test was used to compare mean age of girls and boys. Due to the exploratory nature of our study, no other statistical test to compare groups were performed.

### Reporting and ethical considerations

We adhered to the RECORD guidelines for the reporting of studies using electronic health records.^30^ RPCD is a pseudonymized, opt-out database of GP records. RPCD data are stored confidentially on a local server of ErasmusMC. Under Dutch GDPR law, our study does not require ethical approval. Our study was approved by the RPCD scientific steering committee (project-number 2020-013).

## Results

### Study population

The cohort consisted of 21,717 children (0–17 years, 49.2% female) registered in 17 general practices that employed a YMHPN during the study period. Our algorithm identified 576 possible relevant records (see eFigure 1: Flowchart) which were included in the next step of manual screening. Of these 576 records, 42 records were excluded because they did not concern a YMHPN delivering mental care. In 52 records, the YMHPN assisted the GP (e**.**g**.** discussing cases) but no consultations with child or family took place. In 54 records, the GP clearly advised a child or its caregivers to make an appointment with the YMHPN, but appointments were either not planned or cancelled. In 53 records, GPs noted the possibility to involve the YMHPN, but it was unclear from the record to what extend the GP actually recommended to consult the YMHPN.

The remaining 375 children had one or more consultations with a YMHPN for psychosocial problems and were included in our analysis. These 375 children had a mean age of 12.9 years (SD 3.8) and 59.2% was female (Table 1). Girls consulting a YMHPN were on average older than boys (13.6 vs 12.0 years, *p* < 0.001).

**Table 1:** Treatment trajectories with a YMHPN per sex and age group

	All children consulting a YMHPN *N* = 375	0–6 years *N* = 30	7–12 years *N* = 130	13–17 years *N* = 215	Girls *N* = 222	Boys *N* = 153
Mean age (SD)	12.9 (3.8)	n.a.	n.a.	n.a.	13.6 (3.7)	12.0 (3.8)
Female (%)	59.2%	40.0%	46.9%	69.3%	n.a.	n.a.
Median number of consultations (IQR)	4 (2–7)	3 (1.25–5)	3 (2–6)	4 (2–8)	4 (2–7)	3 (2–6)
≥1 External referral for psychosocial problem (%)^a^	51.7%	43.3%	59.2%	48.4%	49.6%	54.9%
Referral to (%) Mental health care services Other services^b^	42.4% 17.3%	30.0% 13.3%	43.1% 28.5%	43.7% 11.1%	39.2% 18.5%	47.1% 15.7%

^a^Some children received referrals for both mental health services and others services. ^b^Either the community-based support team or the centre for youth and family.

### Type of psychosocial problems

Children consulted YMHPNs with a large variety of psychosocial problems. The three most common ICPC codes describing the psychosocial problem of the child were P22 (‘Other worries about child’s behaviour’, 13.1%), P74 (‘Anxiety disorder’, 8.5%) and P03 (‘Down/Depressive feelings’, 6.9%). The most common code (P22 ‘Other worries about child’s behaviour’) is typically used for a range of worries (e**.**g**.** problems functioning in school, behavioural problems at home). Table 2 lists the 15 most commonly coded problems with the corresponding percentage of children that were eventually referred. The psychosocial problem that most often (91.7%) led to referral was P76 (‘Depressive disorder’).

**Table 2:** Common psychosocial problems managed by YMHPNs and referral rate per problem

	ICPC Code	Code description	Number of children (% of total)^a^	Referral rate per ICPC code^b^
1	P22	Other worries about child’s behaviour	49 children (13.1%)	69.4%
2	P74	Anxiety disorder	32 children (8.5%)	59.4%
3	P03	Down/depressive feelings	26 children (6.9%)	65.3%
4	P29	Other psychiatric symptoms/complaints	26 children (6.9%)	42.3%
5	P01	Anxious/nervous/tensed feelings	23 children (6.1%)	52.3%
6	P21	Attention deficit-/hyperactivity disorder	23 children (6.1%)	56.5%
7	P99	Other psychiatric disorders including autism	14 children (3.7%)	57.1%
8	P76	Depressive disorder	12 children (3.2%)	91.7%
9	P02	Crisis/temporary stress reaction including post-traumatic stress disorder	12 children (3.2%)	33.3%
10	P04	Irritable/angry feeling/behaviour	10 children (2.7%)	50.0%
11	P20	Memory/concentration/orientation disorders	10 children (2.7%)	80.0%
12	Z20	Relationship problem with parents/family	9 children (2.4%)	33.3%
13	P23	Other worries about adolescent’s behaviour	9 children (2.4%)	44.4%
14	Z25	Problems as a consequence of (sexual) violence	9 children (2.4%)	44.4%
15	A80	Trauma/injury	9 children (2.4%)	25.0%

^a^A treatment trajectory by the YMHPN is coded with an ICPC code to describe the type of problem of the child. The proportion represents the percentage of children out of the total sample of *N* = 375 who were coded with a certain ICPC code. ^b^The percentage of children that were eventually referred to external health care providers per ICPC code.

### Treatment trajectories

Children had a median of 4 (IQR 2–7) consultations with the YMHPN during their treatment trajectory. In 44.8% of trajectories, the child missed one or more of their appointments due to no show or last-minute cancellation. In 51.7% of all trajectories, the YMHPN wrote one or more referrals for additional care. Of these first referrals, 44.3% was accepted and 26.8% was rejected. In 28.9% of first referrals, it was unclear from the record whether the referral was accepted. Table 1 summarizes the treatment trajectories per sex and age group (see Supplementary Table S5 for details). There were no large differences in treatment trajectories between sex and age groups. Referral rate to mental health services seemed somewhat more common in boys than in girls (47.1% vs 39.2%).

### End of treatment trajectories

In 22.4% of cases, the child or caregivers decided together with the YMHPN to end treatment sessions because no other consultations were deemed necessary. In 13.3% of cases, the child dropped out of the treatment trajectory. In 52.0% of cases, the trajectory ended with a referral. In 4.8% of cases, the trajectory with the YMHPN was not yet finished after follow up (range 183–365 days). In 7.5% of cases, the YMHPN stopped the treatment trajectory, or it was unclear from the record how the trajectory ended. Table 3 shows the end of trajectory per age group and sex, and the median number of consultations with the YMHPN per group. Overall, there were no large differences in outcome of the treatment trajectory between sex and age groups. However, adolescents had a drop-out rate that was more than twice that of younger age categories.

**Table 3:** Outcomes of the treatment trajectory with the YMHPN per sex and age group

Outcome		All children	0–6 years	7–12 years	13–17 years	Girls	Boys
		N = 375	N = 30	N = 130	N = 215	N = 222	N = 153
Child discontinues trajectory (drop-out):	% number of consultations (median, IQR)	13.3% 2 (1–4)	6.7% 2 (1.5–2.5)	8.5% 1 (1–3.5)	17.2% 2 (1–4)	15.8% 2 (1–4)	9.8% 1 (1–4.5)
Treatment trajectory ends in referral	% number of consultations (median, IQR)	52.0% 4 (2–7)	40.0% 3 (1.75–6.75)	59.2% 3 (2–6)	49.3% 4 (2–8)	51.8% 4 (2–7.5)	52.3% 3 (2–6.25)
Patient and YMHPN decide together to end trajectory, no referral takes place.	% number of consultations (median, IQR)	22.4% 4 (2–6)	33.3% 3.5 (1.25–4.75)	19.2% 4 (2–7)	22.8% 4 (2–6)	17.6% 3 (2–5)	29.4% 4 (2–7)
YMHPN declines trajectory because child was already in specialized care	% number of consultations (median, IQR)	1.1% 1 (1–1.25)	6.7% 1 (1–1)	0.8% 2 (2–2)	0.5% 1 (1–1)	1.4% 1 (1–1.5)	0.7% 1 (1–1)
Trajectory with YMHPN is not yet finished after 1 year	% number of consultations (median, IQR)	4.8% 10 (9–11.75)	3.3% 10 (10–10)	2.3% 11 (9.5–15.5)	6.5% 9.5 (9–13.5)	7.2% 10 (9–11.25)	3.9% 11 (8.5–13.5)
End of trajectory is unclear from record	% number of consultations (median, IQR)	6.4% 2 (1–4.25)	10.0% 2 (2–4)	10.0% 2 (1–4)	3.7% 2.5 (1–3.5)	6.3% 2.5 (1.25–5.75)	6.5% 2 (1.25–3.0)

### Type of care delivered by YMHPNs

In the 247 records with complete documentation in the RPCD, YMHPNs reported to have one or more contacts to discuss treatment options with external care providers (e**.**g**.** school, mental health services) in 50.2% of records. YMHPNs recorded using psychoeducation (25.9%) and CBT-based treatment (13.4%) in a minority of cases. In 17.0% of records, the YMHPN provided E-health interventions for psychological problems. Complete details on the activities of YMHPNs and on the used treatment techniques are shown in Table 4.

**Table 4:** Activities of YMHPN during treatment trajectory

	Children with complete medical file *N* = 247	0–6 years *N* = 20	7–12 years *N* = 88	13–17 years *N* = 139	Girls *N* = 135	Boys *N* = 112
YMHPN had one more contacts concerning the child with external care provider.	50.2%	70.0%	52.3%	46.0%	50.4%	50.0%
YMHPN had contact with: School Child protective services Mental health services Other services^a^	14.2% 5.7% 23.5% 28.7%	25.0% 10.0% 20.0% 50.0%	18.2% 3.4% 26.1% 30.7%	10.1% 6.5% 22.3% 24.5%	14.1% 8.1% 21.5% 28.9%	14.3% 2.7% 25.9% 28.6%
Psychoeducation	25.9%	10.0%	23.9%	29.5%	31.9%	18.8%
CBT-based treatment	13.4%	5.0%	14.8%	13.7%	17.8%	8.0%
E-health	17.0%	5.0%	10.2%	23.0%	19.3%	14.3%
Exercises and assignments	40.1%	25.0%	39.8%	42.4%	42.2%	37.5%

^a^Support team and/or centre for youth and family

## Discussion

### Main findings of this study

In our study we evaluated how YMHPNs manage psychosocial problems in children and adolescents within general practice using electronic records from an extensive GP database. In daily practice, YMHPNs are confronted with a large variety of psychosocial problems. Managed children were often in their adolescence (57.3% was between 13 and 17 years) and were more often female than male (59.2% vs 40.8%). In more than half of cases, the YMHPN eventually referred the child for additional care to external services. In almost one in four cases, the YMHPN successfully managed the problem, and no additional referral or intervention was needed. Approximately one in eight children dropped out during the treatment trajectory.

### What is already known on this topic

It has been suggested that integration of child mental health services into primary care, leads to improved access and quality of mental health care.^31^ A systematic review showed beneficial effects (e**.**g**.** improved health outcomes of affected children) of integrating child mental health services in primary care.^32^ The included studies used different approaches to integrate mental health care into primary care and are mostly not directly comparable to our study design. One of the included studies, however, used a similar approach to our study.^33^ It investigated a nurse-led psychosocial intervention within primary care for children with behavioural problems as compared to usual care. Children treated in primary care with the nurse-led intervention completed treatment more often and showed better, albeit modest, clinical improvement than children receiving usual care.^33^

There are some indications that the introduction of YMHPNs within general practices led to more identification of psychosocial problems without reducing the number of referrals to specialized care.^16^ An earlier study showed that 55% of children managed by a YMHPN were eventually referred to specialized services.^17^

### What this study adds

The goal of this study was to describe characteristics and the type of problems of children consulting YMHPNs, and how YMHPNs managed these problems. We found that children consulting a YMHPN were often in their adolescence, and were more often girl than boy. This findings are in line with the occurrence of mental health problems which increases with increasing age peaking in adolescence.^34^ Additionally, mental health problems tend to be more common in boys during early childhood, while girls are more often affected during adolescence.^35^^,^^36^

YMHPNs appear to manage a wide range of mental health problems, and treatment trajectories were usually relatively short (median of 4 consultations). This aligns with earlier findings showing that the majority of treatment trajectories last 8 weeks or less.^37^ Besides a variety of techniques and treatment approaches, in a minority of patients, the YMHPN reported to use CBT-based interventions. CBT is a treatment for many mental health problems with the largest evidence base, but it can be time-consuming and therefore less suitable for the general practice setting.^38–40^ Brief CBT-based interventions form a promising alternative for traditional CBT, and may be more suitable general practice.^41^ Future research should investigate the effectiveness of brief psychological interventions (e**.**g**.** CBT-based) that can easily be used by YMHPNs for managing psychosocial problems. To date, the evidence base is rather scarce. Further, also the effectiveness of E-health in primary care should be subject of more research,^42^ as it was common for YMHPNs to use E-health in their management.

One of the presumed benefits of the introduction of the YMHPN was that this would lead to more targeted referrals with less rejections of referrals.^16^ However, in our study, at least 26.8% of initial referrals was rejected by specialized health services. This can be burdensome for affected families. Therefore, future research should focus on how to improve and streamline the referral process from primary care to specialized services.

In our study, we observed that YMHPNs were able to successfully manage or treat 22.4% of children. Thus, the YMHPN might fill a treatment gap for those not requiring specialized care but still in need of some treatment for the observed psychosocial problems. Further, for those children and adolescents who are subsequently referred to specialized care, the YMHPN might fulfill an important bridging role, given the long waiting lists for specialized care.^43^^,^^44^ Only 13.3% of children dropped out during the treatment trajectories with YMHPNs, which can be interpreted as good adherence with the treatment offered by the YMHPN in our study. The low number of treatment drop-outs might be explained by the high level of familiarity with the general practice setting and by the accessibility of treatments within this setting. However, it should be noted that treatment trajectories in our study were relatively short, which could also be a reason for the observed low number of treatment drop-outs.

### Limitations of this study

The use of routinely registered health care data for research has disadvantages. Documentation is often limited and YMHPNs may not always have written down all relevant information. Even more, in 128 of 375 records, the YMHPN used two separate systems for documentation, while we only had access to the GP record but not to the second documentation system. Therefore, these records could not be used to describe the care delivered by YMHPNs. Also, the ICPC codes used for defining the target problem often described the reason for consultation (e**.**g**.** ‘Worries about child’s behavior’), rather than the actual problem of the child (e**.**g**.** ‘Anxiety disorder’). Additionally, YMHPNs did not report specific outcomes of their treatment trajectory in the medical record (e**.**g**.** pre- and post-treatment scores on validated scales). Therefore, it is not possible to quantify the effectivity of the treatment trajectory of the YMHPN. Given the observational nature of our data, it was not possible to compare integrated care delivered by a YMHPN with treatment as usual, which would have been possible in a trial design. Finally, it is important to realize that our study was performed in an urban population with a relatively low economic status.^45^ Hence, our results might not necessarily be generalizable to other populations (e**.**g**.** rural or more affluent).

## Conclusion

YMHPNs working in Dutch general practices managed a wide variety of psychosocial problems. In one in four children, the YMHPN successfully managed the problem, and no additional treatment was needed. Drop-out rates were low indicating good treatment adherence. The broad range of problems managed by the YMHPN, together with a considerable proportion of children not needing additional referral to specialized care after consulting a YMHPN as well as low drop-out rates observed in our study indicate the potential usefulness of the YMHPN as an additional source of treatment for children and adolescents with psychosocial problems.

## Availability of data and material

Due to legal constraints, data are not publicly available and access to the data requires approval by the Governance Board of RPCD.

## Supplementary Material

RECORD_statement_Evaluation_delivered_care_fdae008

## Notes

**Lukas B.M. Koet,** PhD student

**Heike Gerger,** Senior Researcher

**Wilma Jansen,** Senior Researcher

**Patrick J.E. Bindels,** Professor of General Practice

**Evelien I.T. de Schepper,** Senior Researcher
